# Errata

**DOI:** 10.36660/abc.20210812

**Published:** 2021-10-06

**Edição de Dezembro de 2020, vol. 115 (6), págs. 1051-1060**

No Artigo Original “Menor Prevalência e Extensão da Aterosclerose Coronária na Doença de Chagas Crônica por Angiotomografia Coronária”, com número de DOI: https://doi.org/10.36660/abc.20200342, publicado no periódico Arquivos Brasileiros de Cardiologia, 115(6):1051-1060, na página 1059, alterar a informação que não houve financiamento externo para: O presente estudo foi financiado pela Capes. Alterar a informação que não há vinculação acadêmica para: Este artigo é parte de tese de Doutorado de Savio Cardoso pela Universidade de São Paulo.

**Edição de Setembro de 2021, vol. 117 (3), págs. 520-527**

No Artigo Original “Hipertensos Tratados e Avaliados por Telemonitoramento Residencial da Pressão Arterial. Estudo TeleMRPA”, com número de DOI: https://doi.org/10.36660/abc.20200073, publicado no periódico Arquivos Brasileiros de Cardiologia, 117(3):520-527, na página 520, alterar a instituição do Dr. Roberto Dischinger Miranda de: Universidade Federal de São Paulo Escola Paulista de Medicina, São Paulo, SP - Brasil para: Seção Cardiovascular, Disciplina de Geriatria e Gerontologia, Escola Paulista de Medicina, Universidade Federal de São Paulo, São Paulo, SP – Brasil e acrescentar para o Dr. Roberto Dischinger Miranda a instituição Hospital Israelita Albert Einstein, São Paulo - Brasil. Com isso a instituição 7 passou a ser: Escola de Ciências Sociais e da Saúde - Pontifícia Universidade Católica de Goiás, Goiânia, GO – Brasil; a instituição 8 passou a ser: Estratégia de Saúde da Família - Secretaria Municipal de Saúde Campos do Jordão, Campos do Jordão, SP – Brasil; a instituição 9 passou a ser: Procape / MCor, Recife, PE – Brasil e a instituição 10 passou a ser: Centro Universitário CESMAC - Hospital do Coração, Maceió, AL - Brasil

**Edição de Setembro de 2021, vol. 117 (3), págs. 561-598**

No “Posicionamento sobre Diagnóstico e Tratamento da Amiloidose Cardíaca – 2021”, com número de DOI: https://doi.org/10.36660/abc.20210718, publicado no periódico Arquivos Brasileiros de Cardiologia, 117(3):561-598, na página 561, foi incluído o autor Flávio Henrique Valicelli, instituição de número 1, após o autor Carlos Eduardo Rochitte. Na página 564, foi inserido o conflito de interesse: “Nada a ser declarado”, abaixo do nome Fernando Bacal.

