# Supplementary material for: Errata
Source: Arq Bras Cardiol. 2021 Oct 6;117(4):910. [Article in Portuguese] doi: 10.36660/abc.20210812 (PMC8528368; doi:10.36660/abc.20210812)
Supplement: Translation

# Translation: Erratum

**December 2020 Issue, vol. 115 (6), pages 1051-1060**

In Original Article “Lower Prevalence and Severity of Coronary Atherosclerosis in Chronic Chagas’ Disease by Coronary Computed Tomography Angiography” with DOI number: https://doi.org/10.36660/abc.20200342, published in the Journal Arquivos Brasileiros de Cardiologia, 115(6):1051-1060, on page 1059, change the information that there was no external funding to: This study was funded by Capes. Change information that is not associated to: This article is part of the thesis of doctoral submitted by Savio Cardoso from Universidade de São Paulo.

**September 2021 Issue, vol. 117 (3), pages 520-527**

In Original Article “ Treated Hypertensive Patients Assessed by Home Blood Pressure Telemonitoring. TeleMRPA Study”, with DOI number: https://doi.org/10.36660/abc.20200073, published in the Journal Arquivos Brasileiros de Cardiologia, 117(3):520-527, on page 520, change the institution of Dr. Roberto Dischinger Miranda from: Universidade Federal de São Paulo Escola Paulista de Medicina, São Paulo, SP - Brasil to: Seção Cardiovascular, Disciplina de Geriatria e Gerontologia, Escola Paulista de Medicina, Universidade Federal de São Paulo, São Paulo, SP - Brasil and add for Dr. Roberto Dischinger Miranda the institution Hospital Israelita Albert Einstein, São Pauo, SP - Brazil. Thus, institution 7 became: Escola de Ciências Sociais e da Saúde - Pontifícia Universidade Católica de Goiás, Goiânia, GO – Brazil; institution 8 became: Estratégia de Saúde da Família - Secretaria Municipal de Saúde Campos do Jordão, Campos do Jordão, SP – Brazil; institution 9 became: Procape / MCor, Recife, PE – Brazil and institution 10 became: Centro Universitário CESMAC - Hospital do Coração, Maceió, AL – Brazil.

**September 2021 Issue, vol. 117 (3), pages 561-598**

In “Position Statement on Diagnosis and Treatment of Cardiac Amyloidosis – 2021”, com número de DOI: https://doi.org/10.36660/abc.20210718, published in the Journal Arquivos Brasileiros de Cardiologia, 117(3):561-598, on page 561, author Flávio Henrique Valicelli, institution number 1, after the author Carlos Eduardo Rochitte was included. On page 564, the conflict of interest was included: “Nothing to be declared”, under the name Fernando Bacal.
